# Predicting Ectopic Pregnancy Using Human Chorionic Gonadotropin (hCG) Levels and Main Cause of Infertility in Women Undergoing Assisted Reproductive Treatment: Retrospective Observational Cohort Study

**DOI:** 10.2196/17366

**Published:** 2020-04-16

**Authors:** Huiyu Xu, Guoshuang Feng, Yuan Wei, Ying Feng, Rui Yang, Liying Wang, Hongxia Zhang, Rong Li, Jie Qiao

**Affiliations:** 1 Peking University Third Hospital Beijing China; 2 Beijing Children’s Hospital Beijing China

**Keywords:** β-hCG, ectopic pregnancy, intrauterine pregnancy, biochemical pregnancies, IVF/ICSI-ET

## Abstract

**Background:**

Ectopic pregnancy (EP) is a serious complication of assisted reproductive technology (ART). However, there is no acknowledged mathematical model for predicting EP in the ART population.

**Objective:**

The goal of the research was to establish a model to tailor treatment for women with a higher risk of EP.

**Methods:**

From December 2015 to July 2016, we retrospectively included 1703 women whose serum human chorionic gonadotropin (hCG) levels were positive on day 21 (hCG21) after fresh embryo transfer. Multivariable multinomial logistic regression was used to predict EP, intrauterine pregnancy (IUP), and biochemical pregnancy (BCP).

**Results:**

The variables included in the final predicting model were (hCG21, ratio of hCG21/hCG14, and main cause of infertility). During evaluation of the model, the areas under the receiver operating curve for IUP, EP, and BCP were 0.978, 0.962, and 0.999, respectively, in the training set, and 0.963, 0.942, and 0.996, respectively, in the validation set. The misclassification rates were 0.038 and 0.045, respectively, in the training and validation sets. Our model classified the whole in vitro fertilization/intracytoplasmic sperm injection–embryo transfer population into four groups: first, the low-risk EP group, with incidence of EP of 0.52% (0.23%-1.03%); second, a predicted BCP group, with incidence of EP of 5.79% (1.21%-15.95%); third, a predicted undetermined group, with incidence of EP of 28.32% (21.10%-35.53%), and fourth, a predicted high-risk EP group, with incidence of EP of 64.11% (47.22%-78.81%).

**Conclusions:**

We have established a model to sort the women undergoing ART into four groups according to their incidence of EP in order to reduce the medical resources spent on women with low-risk EP and provide targeted tailor-made treatment for women with a higher risk of EP.

## Introduction

Ectopic pregnancy (EP) is the leading cause of maternal morbidity and mortality during the first trimester, accounting for 5% to 10% of all maternal deaths [1]. Moreover, the incidence of EP is 2 to 3 times higher in pregnancies resulting from assisted reproductive technology (ART) than in natural pregnancies [2]. It is well acknowledged that the circulating human chorionic gonadotropin (hCG) level in early pregnancy aids in diagnosis of EP before any gestational sac can be visualized through ultrasonography. However, a meta-analysis has suggested that the efficacy of a single serum hCG test to predict an EP is low; an hCG ratio strategy—which is the ratio between two successive time points of hCG concentration—has better sensitivity, while regression models have better specificity but need further improvement and validation [3]. To date, there is no acknowledged mathematical model for predicting EP in women undergoing in vitro fertilization (IVF) or intracytoplasmic sperm injection (ICSI) and embryo transfer (ET) treatment. Thus, a significant amount of time and resources are spent in reproductive centers on monitoring women with early pregnancies to identify EP in time to prevent its complications. Early tests for assuring the location of gestational sacs have significant cost burdens on patients and centers.

The aim of this study was to establish such a model to rank the women undergoing IVF/ICSI-ET treatment into a few groups according to the incidence of EP. The goals are to reduce medical resources spent on the low-risk EP group, provide more targeted tailor-made treatment for women at a high risk of EP, and further improve the detection rate for this adverse outcome.

## Methods

### Subjects

**Figure 1:**
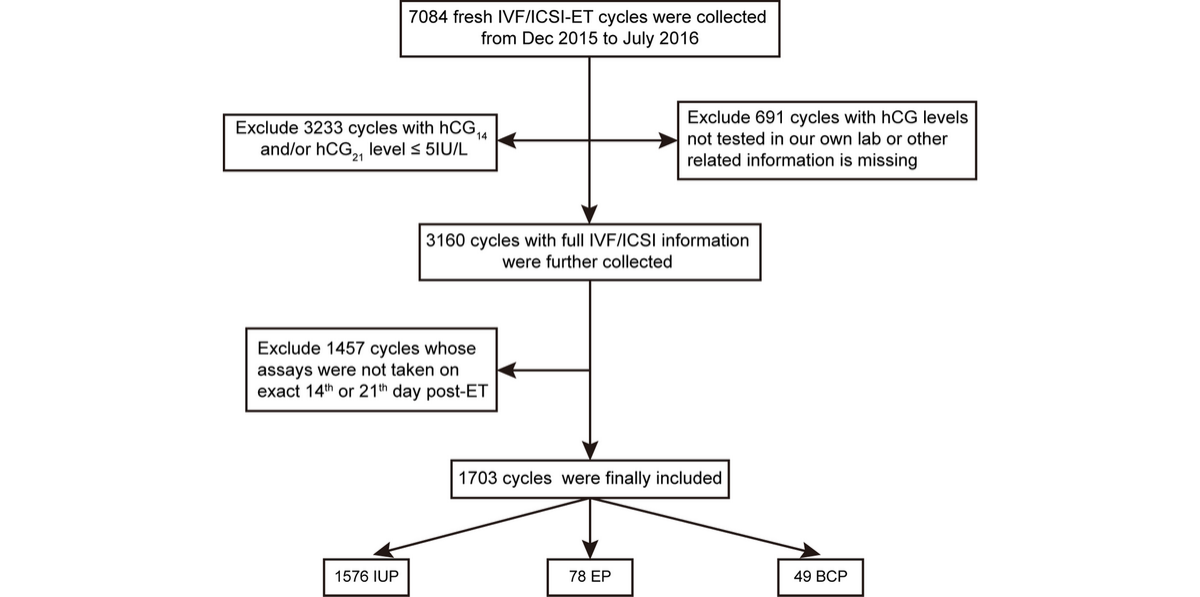
Flowchart of the data selection strategy. hCG14 and hCG21: serum hCG levels on days 14 and 21 post–embryo transfer; EP: ectopic pregnancy; ET: embryo transfer; IUP: intrauterine pregnancy; BCP: biochemical pregnancy.

This was a retrospective observational cohort study performed from December 2015 to July 2016. Datasets of all fresh ET cycles were recorded. Data were entered into a database by the clinical support staff. The database was used to collect basic and clinical characteristics of patients including age, body mass index, baseline sex hormone levels, main causes of infertility, endometrial thickness on the day of hCG used for triggering ovulation, details of ovarian stimulation protocols, insemination method, date of insemination, date of ET, numbers of ETs, date of hCG examination, serum concentrations of hCG, fertilization results, and pregnancy types, including EP, biochemical pregnancy (BCP), and intrauterine pregnancy (IUP). The inclusion criteria were (1) serum hCG level >5 IU/L on days 14 (hCG_14_) and 21 post-ET (hCG_21_); (2) hCG examinations were tested in our own lab (the same platform); and (3) hCG levels were tested exactly on day 14 or 21 post-ET. Of these, 1703 cycles were selected. The cycles were further divided into three outcome groups: EP, IUP, or BCP. A flowchart of this process is shown in Figure 1. During the study period, 7084 fresh IVF/ICSI-ET cycles were enrolled in our study. Of these, 1703 cycles that met the inclusion criteria were selected. There were 1576 (92.54%) women with an IUP, 78 (4.58%) with an EP, and 49 (2.88%) with a BCP. The basic and clinical characteristics in relation to different pregnancy outcomes were shown in Table 1.

**Table 1:** Basic and clinical characteristics in related to different pregnancy outcomes.

Characteristic		Intrauterine pregnancy, (n=1576)	Ectopic pregnancy, (n=78)	Biochemical pregnancy, (n=49)
Age in years, mean (quartile)		32 (29-35)	32 (29-35)	32 (30-35)
Body mass index (kg/m^2^), mean (quartile)		22.1 (20.3-24.5)	22.5 (19.5-24.5)	22.6 (20.1-25.5)
**Cause of infertility, n (%)**				
	Male infertility	530 (33.6)	16 (20.5)	24 (49.0)
	Endometriosis	46 (2.9)	1 (1.3)	2 (4.1)
	Anovulatory infertility	81 (5.2)	9 (11.5)	5 (10.2)
	Tubal factor	639 (40.5)	35 (44.9)	15 (30.6)
	Unexplained and others	280 (17.8)	17 (21.8)	3 (6.1)
Retrieved oocytes, mean (quartile)		10 (7-14)	10 (7-13)	10 (6-14)
**ET^a^ on day 3 or day 5 postinsemination, n (%)**				
	Cleavage	1540 (97.7)	76 (97.4)	46 (93.9)
	Blastocyst	36 (2.3)	2 (2.6)	3 (6.1)
**Embryos transferred, n (%)**				
	1	105 (6.7)	5 (6.4)	5 (10.2)
	2	1471 (93.3)	73 (93.6)	44 (89.8)
hCG_14_^b^, mean (quartile)		827 (524-1300)	186 (103-289)	139 (71-300)
hCG_21_^c^, mean (quartile)		15,570 (9954-22,626)	1870 (815-3107)	95 (27-275)
Ratio of calculated 48-hour rising, mean (quartile)		2.3 (2.1-2.4)	1.9 (1.5-2.4)	0.9 (0.6-1.1)
hCG_21_/hCG_14_, mean (quartile)		17.5 (13.8-22.0)	10.3 (4.1-20.4)	0.7 (0.2-1.4)

^a^ET: embryo transfer.

^b^hCG_14_: serum level of human chorionic gonadotropin on 14th day post–embryo transfer.

^c^hCG_21_: serum level of human chorionic gonadotropin on 21st day post–embryo transfer.

### In Vitro Fertilization/Intracytoplasmic Sperm Injection–Embryo Transfer Protocols

The ovarian stimulation protocols used in our center include a gonadotrophin releasing hormone (GnRH) antagonist protocol, a GnRH agonist long protocol, a GnRH agonist short protocol, and mild stimulation protocols, as described previously [4,5]. Briefly, when two or more leading follicles reached a diameter of 18 mm as measured by ultrasonography, 5000 to 10000 IU recombinant hCG was administered. Transvaginal ovum collection was performed 36 to 38 hours later. The collected oocytes were fertilized by IVF or ICSI. After 3 or 5 days of culture, the embryos were either transferred freshly to the mother or cryopreserved. Luteal support was carried out from the day of oocyte retrieval. It is generally recommended to use vaginal administration. In the case of patients with vaginal bleeding, oral plus muscle injection are recommended. At 8 to 10 gestational weeks, if there is no bleeding or signs of threatened early miscarriage, luteal support could be terminated.

### Pregnancy Outcomes

An IUP was defined as one or more intrauterine gestational sacs detected by transvaginal sonography (TVS) at 30 or 37 days after embryo transfer. As the heartbeat is not necessarily present on the 30th or 37th day post-ET, as long as the gestational sac is seen within the uterus on the 30th or 37th day post-ET it is an IUP, which includes a certain proportion of first-trimester miscarriage. An EP was diagnosed by visualization of one or more gestation sacs outside the uterus detected by TVS. A BCP was indicated by a temporary rise of serum hCG without gestational sacs inside or outside the uterus detected by TVS.

### Beta–Human Chorionic Gonadotropin Assays

The serum β-hCG level of each patient was assessed from December 2015 to July 2016 using an Access UniCel DxI 800 chemiluminescence system and an Access total β-hCG assay kit (both Beckman Coulter Inc), standardized to the highly purified World Health Organization 5th International Standard for hCG. Quality controls used were the Lyphochek trilevel Immunoassay Plus Controls (catalogue 370; lot number 40320; Bio-Rad Laboratories). The interassay variation was 7.9% in low-level Bio-Rad immunoassays and controls, 7.4% in mid-level controls, and 4.1% in high-level controls.

### Statistical Analysis

Normally distributed variables were presented as mean and standard deviation. Nonnormally distributed variables were presented as median and quartile. Before further analysis, a generalized additive model was used to explore the suitable function between explanatory variables and outcome. The outcome variables were classified into three subgroups: EP, IUP, and BCP. Multinomial logistic regression was used because there were more than two outcome variables. Before analysis, the dataset was partitioned into a training set and a validation set at the proportion of 0.75:0.25, and multinomial logistic regression was performed on the training set to establish the prediction model. Specifically, the hCG_21_ and hCG_21_/hCG_14_ ratio were entered as quadratic forms, and the cause of infertility was treated as a dummy variable with reference to male-factor infertility. Akaike’s information criterion (AIC) and Schwarz-Bayesian information criterion (SBIC) were used to compare various models to determine the best-fitting model; the model having the smallest AIC and BIC values was preferred. The model was then applied to the validation set, and the areas under the receiver operating curve (AUC) and misclassification rates were calculated for model evaluation. To build a more targeted predictive model, according to the incidence of EP, we partitioned cases into 12 groups based on the prediction probability of EP and BCP in each group, using the actual outcome proportions of the three categories. An exact (Clopper-Pearson) confidence limits or Wald confidence limits method was used to calculate the 95% confidence intervals of EP incidence. The data were analyzed with JMP Pro version 14.0 software (SAS Institute Inc), and a 2-sided *P*-value of <.05 was considered statistically significant.

## Results

### Univariate Multinomial Logistic Regression to Determine Relationships Between Independent Variables and Different Pregnancy Outcomes

As the early pregnancy outcome was an EP, IUP, or BCP, we used univariate multinomial logistic regression to test the relationships between each independent variable and the outcome variable. Considering the strong correlation between hCG_14_ and hCG_21_ (*R*^2^=.74)—which is an indication of collinearity—the serum levels of hCG_14_ and hCG_21_ could not be included in the prediction model simultaneously, so we only included the hCG_21_ level in further analysis. First, a generalized additive model was used to explore the relationship between continuous independent variables and the dependent variable. The hCG_21_ and hCG_21_/hCG_14_ ratio were quadratically related to the dependent variable. Therefore, the hCG_21_ and the hCG_21_/hCG_14_ ratio was included as a quadratic term in the analysis. Univariate analysis showed that the cause of infertility, hCG_21_, hCG_21_^2^, hCG_21_/hCG_14_, (hCG_21_/hCG_14_)^2^, and cleavage or blastocyst embryo transfer were statistically significant, as shown in Multimedia Appendix 1.

### Multivariate Multinomial Logistic Regression to Establish the Predictive Model

The independent variables identified in the univariate analysis were further examined by multivariate multinomial logistic regression. Cleavage or blastocyst embryo transfer was not of significance in predicting pregnancy outcomes because after removing this independent variate, the SBIC and AIC were reduced from 385.44 to 371.83 and 283.06 to 279.64, respectively. To distinguish EP and non-EP, we explored the cutoff value of the predictive model. The default cutoff value of the software is 0.5, which can be adjusted with reference to the prevalence of EP. Based on the incidence of EP in our data and referring to Van Calster’s [6] cutoff for predicting EP, we found that a cutoff value of 5% might be the best distinction for our model. The final independent variates included in the multivariate multinomial logistic regression model for predicting the different pregnancy outcomes were the cause of infertility, hCG_21_, hCG_21_^2^, hCG_21_/hCG_14_, and (hCG_21_/hCG_14_)^2^, as indicated in Multimedia Appendix 2. To illustrate this, if the value of estimation is positive, the probability of EP or BCP increases with an increase in the predictive factor, and if the value of estimation is negative, the probability of EP or BCP increases with a decrease in the predictive factor. Furthermore, concerning odds ratios referring to the main causes of infertility, a woman with anovulatory-induced infertility had higher odds of an EP, with an odds ratio of 7.24 (1.66-31.63); while a woman in a couple with male-factor infertility had higher odds of having a BCP, with an odds ratio of 20 compared with tubal infertility Multimedia Appendix 2.

### Evaluation of the Model

**Figure 2:**
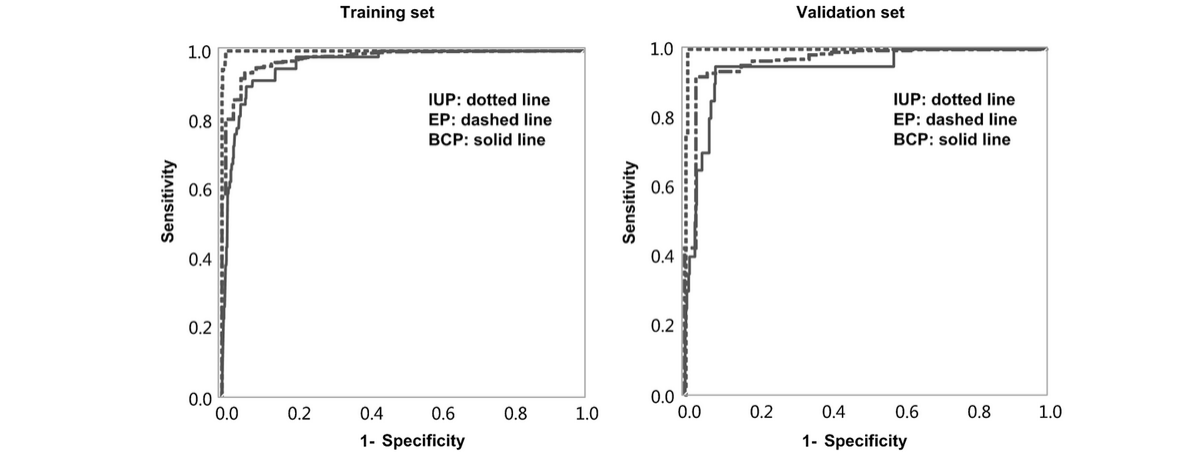
Sensitivity and specificity of the model in the training and validation sets.

The ability of the model to predict one outcome versus the other two outcomes in the training and validation sets was evaluated by the AUC analysis and misclassification rate, as shown in Table 2. The AUC values for IUP, EP, and BCP were 0.978, 0.962, and 0.999 in the training set, respectively, and 0.963, 0.942, and 0.996 in the validation set, respectively. The misclassification rates were 0.038 and 0.045 in the training and validation sets, respectively. The sensitivity and the specificity of the models in the two sets are shown in Figure 2. Table 3 displays the performance of our data in detail. For example, in the training set, a total of 1172 predicted cases of IUP turned out to be actual cases, accounting for 99.15% of the total. However, only 21 predicted EPs turned out to be actual EPs, accounting for only 36.21% of the total. Therefore, we tried to explore why so many EPs could not be predicted.

**Table 2:** The performance of the predicting model.

Datasets	Area under the receiver operating curve			MR^d^	
	IUP^a^, (n=1576)	EP^b^, (n=78)	BCP^c^, (n=49)		
Training set	0.978	0.962	0.999		0.038
Validation set	0.963	0.942	0.996		0.045

^a^IUP: intrauterine pregnancy

^b^EP: ectopic pregnancy.

^c^BCP: biochemical pregnancy.

^d^MR: misclassification rate

**Table 3:** The predicted and actual occurrence of different pregnancy outcomes in our data.

Actual pregnancy outcomes	Predicted pregnancy outcomes, n (%)					
	Training set			Validation set		
	IUP^a^, (n=1208)	EP^b^, (n=31)	BCP^c^, (n=38)	IUP, (n=398)	EP, (n=13)	BCP, (n=15)
IUP	1172 (97.0)	9 (29.0)	1 (2.6)	387 (97.2)	5 (38.5)	2 (13.3)
EP	35 (2.9)	21 (67.7)	2 (5.3)	11 (2.8)	8 (61.5)	1 (6.7)
BCP	1 (0.1)	1 (3.2)	35 (92.1)	0 (0)	0 (0)	12 (80.0)

^a^IUP: intrauterine pregnancy

^b^EP: ectopic pregnancy.

^c^BCP: biochemical pregnancy.

**Figure 3:**
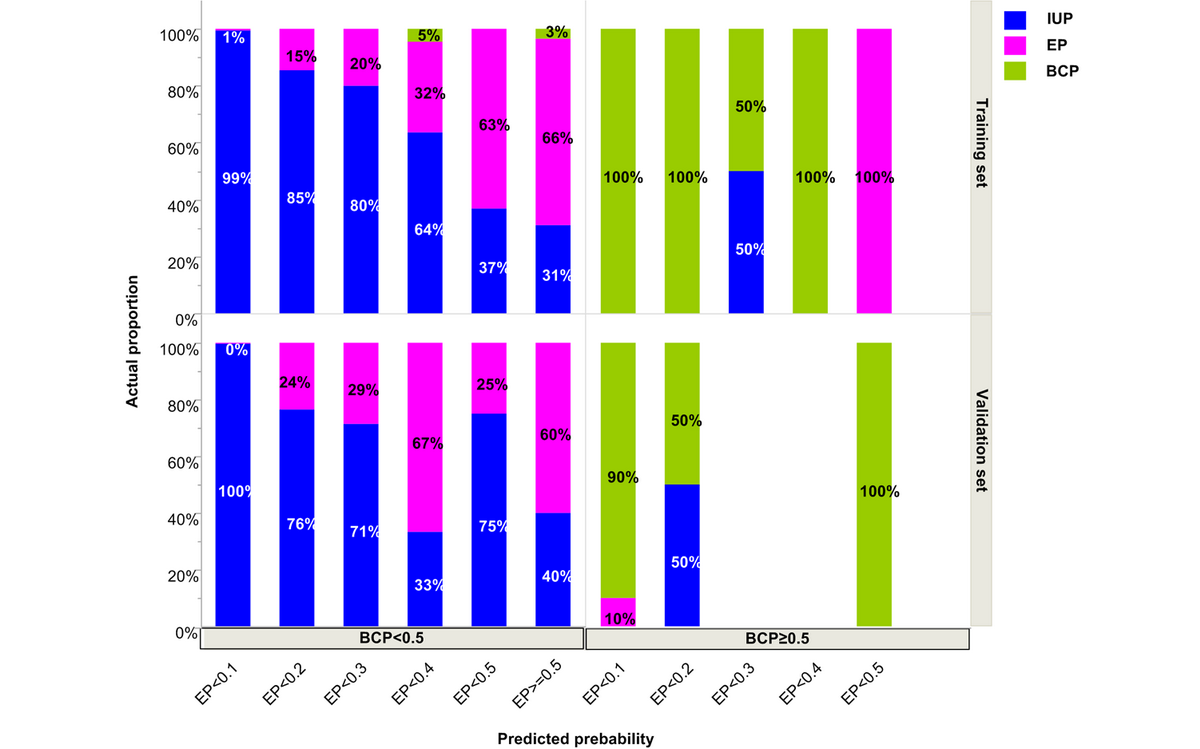
Classifying the population into subgroups according to the predicted probabilities of IUP, EP, and BCP using a training set and a validation set of data. IUP: intrauterine pregnancy; EP: ectopic pregnancy; BCP: biochemical pregnancy.

For this, we further explored the grouping method according to the predicted probabilities of pregnancy outcomes. Because the sum of the predicted probabilities of IUP+EP+BCP=1, if two predicted probabilities of EP and BCP are known, the other one is known. So, we divided the whole population into more groups based on the predicted probabilities of EP and BCP. As shown in Figure 3, the probability of EP was divided into 6 groups of <0.1, 0.1 to <0.2, 0.2 to <0.3, 0.3 to <0.4, 0.4 to <0.5, and ≥0.5. BCP was divided into two groups, with probabilities of <0.5 and ≥0.5. Thus, 12 (6×2) groups were formed, as indicated in Figure 3. The whole population was further divided into 4 groups, as shown in Table 4. The first group was the low-risk EP group with a predicted EP probability of <0.1 and a predicted BCP probability of <0.5. The low-risk EP population accounted for 85.7% of the whole population, and the actual incidence of EP in this group was 0.52% (95% CI 0.23%-1.03%). The second group was the predicted BCP group, with an incidence of EP of 5.79% (95% CI 1.22%-15.95%), which was significantly higher than that of the low-risk EP group. Women in this group also had higher chances of undergoing spontaneous abortion. The third group was the indeterminate group with a predicted EP probability of 0.1 to <0.5 and BCP of <0.5 and an incidence of EP of 28.32% (95% CI 21.10%-35.53%), significantly higher than the incidences in the first and second groups. The fourth group was the high-risk EP group (predicted EP group with predicted EP probability of ≥0.5), with a predicted incidence of EP of 64.11% (95% CI 47.22%-78.81%).

**Table 4:** Classification according to the incidence of ectopic pregnancy (n=1703).

Group	Predicted probability	n (%)	Incidence of EP^a^ (%)	Number of actual cases		
				IUP^b^	EP	BCP^c^
1: EP low risk	Prob_EP_<0.1 & Prob_BCP_<0.5	1460 (85.73)	0.52 (0.23-1.03)	1453	7	0
2: predicted BCP	Prob_BCP_≥0.5	52 (3.05)	5.79 (1.21-15.95)	2	3	47
3: gray zone	0.1≤Prob_EP_<0.5 & Prob_BCP_<0.5	152 (8.93)	28.32 (21.10-35.53)	108	43	1
4: EP high risk	Prob_EP_≥0.5	39 (2.29)	64.11 (47.22-78.81)	13	25	1

^a^EP: ectopic pregnancy.

^b^IUP: intrauterine pregnancy

^c^BCP: biochemical pregnancy.

## Discussion

### Principal Findings

Here, through the multivariate multinomial logistic regression method, we have established a mathematical model to predict the probability of having an IUP, EP, or BCP in pregnant women subjected to ART using predictors of hCG_21_, ratio of hCG_21_/hCG_14_, and main cause of infertility. We further classified the whole population into four subgroups according to the incidence of EP in each group in order to rearrange our clinical routine to reduce medical resources spent on women with a low risk of EP and provide more targeted tailor-made treatments for women with a higher risk of EP.

Considering that current routine clinical examinations cannot diagnose EP in early pregnancy, the routine in our reproductive center for a woman undergoing IVF/ICSI-ET treatment is to measure serum hCG levels around day 14 and 21 post-ET and then take two TVS tests to confirm the location of the gestational sac on day 30 and 37 post-ET, with sometimes even another test on day 44 post-ET. Based on the good predictive effect of our model, we are currently developing this regression model into computer software to better manage women in early pregnancy according to their risk of EP. To be specific, for the low-risk EP group (accounting for 85.73% of the whole population), we are considering reducing the frequency of TVS tests to one on day 30 post-ET. For the predicted high-risk EP group, with incidence of EP of 64.11% (95% CI 47.22%-78.81%), an immediate TVS examination is recommended after the hCG_21_ test. For the grey zone group, with incidence of EP of 28.32% (95% CI 21.10%-35.53%), the original frequency of two TVS visits is recommended. For the predicted BCP group, although the incidence of EP is significantly higher than that in the low-risk EP group, the likelihood of having a spontaneous abortion is also high and these women can be treated as belonging to the low-risk EP group.

The acknowledged M4 model for predicting EP in pregnancies of the unknown location (PUL) population [7] has been used in several hospitals and has successfully reduced the number of visits, blood tests, and scans in women of early gestational age with a PUL [6]. We hope that the clinical application of our model could first reduce the TVS visit in the general ART population, second, identify the women with a high risk of EP and give them immediate treatment, and third, leave similar or reduced proportion of undiagnosed cases of EP after the time point of day 37 post-ET compared with the clinical routine of 2 TVS visits.

An hCG ratio strategy was reported to have a better sensitivity in predicting EP compared with a single serum hCG level [3,8]. Dart et al [9] reported that using an hCG increase <66% to predict EP had a sensitivity of 74%, while an hCG decrease <50% had a better sensitivity of 80%. Bignardi et al [10], using an hCG ratio of <1.66 to predict EP, reported a sensitivity of 85%, but when they increased the cutoff value to an hCG ratio of <2, the sensitivity increased to 92% but the specificity was not satisfactory. The use of multivariate models to predict EP gave better specificity [8]. According to a study by Condous et al [7], specificity using a logistic regression multivariate model of hCG ratio (hCG at 48 hours/hCG at 0 hours) to predict EP was 87%. More complicated models achieved better specificity in women with a PUL [6,8,11]. However, they were not applied to women undergoing IVF/ICSI-ET, and the criteria for the included populations were highly heterogeneous [3].

The idea of predicting EP using multinomial logistic regression was actually derived from the work of Condous et al [7] on predicting EPs in women with a PUL [6,11]. There were two different features in our study. First, the enrolled populations in those studies only included women with a PUL [6,11]; however, we included all the IVF/ICSI-ET cycles during the study period. Second, we further classified the population into four groups instead of two (EP high- and low-risk groups) [6] according to the incidence of EP in each group. Grouping the whole population into four groups instead of two is very useful. For example, according to the multinomial logistical model, a woman is predicted to have an IUP, with a predicted IUP probability of 51%, predicted EP probability of 39%, and predicted BCP probability of 10%. Meanwhile, another woman is also predicted to have an IUP, with a predicted IUP probability of 98%, predicted EP probability of 1%, and predicted BCP probability of 1%. However, their risk of having an EP is significantly different. Our grouping method of classifying the whole population into four groups according to the incidence of EP in each group effectively avoids this problem.

Tubal factor infertility was reported to be the most prominent risk factor for EP after IVF/ICSI-ET treatment [12-14]. However, this was not significantly linked to EP in the study of Condous et al [11] and our study. This might have been because of differences in the enrolled populations and different pretreatment protocols in different ART centers. In our data, couples with male-factor infertility had a high probability of BCP, and those with anovulatory infertility had a high probability of having an EP (Multimedia Appendix 2).

The prevalence of EP per clinical pregnancy in fresh IVF/ICSI-ET cycles was reported to be 4.6% [15], while in our data, the incidence of EP is 4.6% in hCG_21_ positive pregnancies. In our data, clinical pregnancies accounted for more than 90% of all hCG_21_ positive pregnancies between 2016-2018, which means that while the differences of EP incidence between ours and Huang et al [15] is similar, our EP incidence is a little bit more than theirs, which may be induced by random error when including the subjects. Another reason for the slight differences may be that the high-risk EP group is relatively easier to identify by clinicians, and these patients are more prone to stick to our clinical practice of taking the blood test for hCG exactly on day 14 and 21 post-ET; thus, the included proportion of EP in our study is a little bit more than the whole fresh IVF/ICSI-ET population. In addition, in our reproductive center, the incidences of EP per fresh embryo transferred cycles in 2016, 2017, and 2018 were 1.0%, 1.0%, and 1.1%, respectively, which lies in the range of reported 1.0% to 2.0% per fresh embryo transferred cycles in the United States in 2001-2011 [16].

### Limitations

A major limitation in our study is the lack of confirmed efficacy of our model compared with the traditional method; we aim to design a randomized controlled study for this. The outcome measurement is the incidence of EP after the 37th day post-ET. We sought to determine if the incidence of EP detected after that time point in the group using our model is comparable or better than in the group using the traditional clinical routine. Second, although our groups 2 to 4 (Table 4) included 91% of actual cases of EP (71/78), there were still several left undiscovered in the low-risk EP group (group 1), which needs the TVS examination for an accurate diagnosis. Third, whether our software can be used in natural conception pregnancies is still unknown. However, for those women with known date of last menstrual period and regular menstrual cycles of known length, the calculated date equivalent to 14th and 21st day post-ET can be deduced, and such women might be potential users of our model.

### Conclusion

A significant amount of time and resources are spent in ART centers on monitoring women with early pregnancies to identify EP in time to prevent its complications. Early tests for assuring the location of gestational sacs have significant cost burdens on patients and centers. In our study, we established a mathematical model for predicting EP according to the incidence of EP. According to our model, we have sought to rearrange our clinical routine to reduce the medical resources spent on women with low EP risk and provide targeted tailor-made treatment for women with a higher risk of EP. We hope that this method can enable the reasonable use of limited medical resources and improve the efficiency in the management of pregnancies in woman undergoing IVF/ICSI-ET treatments.

## Appendix

Multimedia Appendix 1 Univariate logistic analysis to assess the effect of the independent variables on different pregnancy outcomes.

Multimedia Appendix 2 Multiple logistic analysis to establish the prediction model.

## Abbreviations

AIC: Akaike’s information criterion
ART: assisted reproductive technology
AUC: areas under the receiver operating curve
BCP: biochemical pregnancy
EP: ectopic pregnancy
ET: embryo transfer
GnRH: gonadotrophin releasing hormone
hCG: human chorionic gonadotropin
hCG14: day 14 post–embryo transfer
hCG21: day 21 post–embryo transfer
ICSI: intracytoplasmic sperm injection
IUP: intrauterine pregnancy
IVF: in vitro fertilization
PUL: pregnancies of unknown location
SBIC: Schwarz-Bayesian information criterion
TVS: transvaginal sonography
