# Supplementary material for: Predicting Ectopic Pregnancy Using Human Chorionic Gonadotropin (hCG) Levels and Main Cause of Infertility in Women Undergoing Assisted Reproductive Treatment: Retrospective Observational Cohort Study
Source: JMIR Med Inform. 2020 Apr 16;8(4):e17366. doi: 10.2196/17366 (PMC7193436; doi:10.2196/17366)
Supplement: Multimedia Appendix 1 [file medinform_v8i4e17366_app1.docx]

| Multimedia Appendix 1. Univariate logistic analysis to assess the effect of the independent variables on different pregnancy outcomes. | | | | | | | | | | | |
| --- | --- | --- | --- | --- | --- | --- | --- | --- | --- | --- | --- |
|  | EP VS IUP | | | | |  | BCP VS IUP | | | | |
|  | Parameter estimation | Standard error | Wald χ2 | OR (95%CI) | *P* value |  | Parameter estimation | Standard error | Wald χ2 | OR (95%CI) | P value |
| Age | -0.0162 | 0.0323 | 0.2516 | 0.9839(0.9236-1.0482) | 0.616 |  | -0.0111 | 0.04 | 0.0769 | 0.989(0.9144-1.0696) | 0.782 |
| BMI | -0.0048 | 0.041 | 0.0138 | 0.9952(0.9184-1.0785) | 0.906 |  | 0.038 | 0.0463 | 0.6738 | 1.0387(0.9486-1.1374) | 0.412 |
| Cause 2 *vs.*1 * | -0.0675 | 1.0534 | 0.0041 | 0.9347(0.1186-7.3681) | 0.949 |  | 0.3574 | 0.7692 | 0.2159 | 1.4296(0.3166-6.4561) | 0.642 |
| Cause 3 *vs.*1 * | 1.1603 | 0.5132 | 5.1129 | 3.1909(1.167-8.7248) | 0.024 |  | 0.4866 | 0.5732 | 0.7207 | 1.6268(0.5289-5.0032) | 0.396 |
| Cause 4 *vs.*1 * | 0.4783 | 0.3486 | 1.8826 | 1.6133(0.8147-3.1949) | 0.17 |  | -0.524 | 0.3831 | 1.8708 | 0.5921(0.2795-1.2547) | 0.171 |
| Cause 5 *vs.*1 * | 0.6374 | 0.4013 | 2.5224 | 1.8916(0.8614-4.1535) | 0.112 |  | -1.5027 | 0.7523 | 3.9893 | 0.2225(0.0509-0.9722) | 0.046 |
| Number of retrieved oocytes | 0.0117 | 0.0284 | 0.1684 | 1.0118(0.957-1.0697) | 0.681 |  | 0.0156 | 0.0351 | 0.1986 | 1.0157(0.9482-1.0881) | 0.656 |
| ET: cleavage vs. blastocyst | 0.5025 | 0.7475 | 0.4519 | 1.6528(0.3819-7.1535) | 0.501 |  | 1.4071 | 0.6353 | 4.9065 | 4.0841(1.1758-14.1865) | 0.027 |
| Number of embryo transferred | -0.0202 | 0.531 | 0.0014 | 0.98(0.3461-2.7747) | 0.97 |  | -0.5127 | 0.5419 | 0.8948 | 0.5989(0.207-1.7323) | 0.344 |
| hCG_21_ | -0.0006 | 0.00007 | 76.163 | —— | <.001 |  | -0.0068 | 0.0015 | 21.476 | —— | <.001 |
| hCG_21_^2^ | 8.50E-09 | 1.94E-09 | 19.299 | —— | <.001 |  | 1.06E-07 | 6.48E-08 | 2.6734 | —— | 0.102 |
| hCG_21_/hCG_14_ | -23.0694 | 2.8938 | 63.5509 | —— | <.001 |  | -37.4855 | 5.5462 | 45.6805 | —— | <.001 |
| (hCG_21_/hCG_14_)^2^ | 4.9022 | 0.664 | 54.5006 | —— | <.001 |  | 7.4963 | 1.3963 | 28.8233 | —— | <.001 |
| Key: EP, ectopic pregnancy; IUP, intrauterine pregnancy; BCP, biochemical pregnancy; 1, male infertility; 2, endometriosis; 3, annovulatory infertility; 4,tubal factor infertility; 5, unexplained infertility and others; ET, embryo transfer. | | | | | | | | | | | |
