# Supplementary material for: Predicting Ectopic Pregnancy Using Human Chorionic Gonadotropin (hCG) Levels and Main Cause of Infertility in Women Undergoing Assisted Reproductive Treatment: Retrospective Observational Cohort Study
Source: JMIR Med Inform. 2020 Apr 16;8(4):e17366. doi: 10.2196/17366 (PMC7193436; doi:10.2196/17366)
Supplement: Multimedia Appendix 2 [file medinform_v8i4e17366_app2.docx]

Multimedia Appendix 2. Multiple logistic analysis to establish the prediction model.

|  | | | |  |  |  |  |  |  |  |  |
| --- | --- | --- | --- | --- | --- | --- | --- | --- | --- | --- | --- |
|  | EP VS IUP | | | | |  | BCP VS IUP | | | | |
|  | Parameter estimation | Standard error | Wald χ2 | OR(95%CI) | *P* value |  | Parameter estimation | Standard error | Wald χ2 | OR(95%CI) | *P* value |
| hCG_21_ | -0.0007 | 0.0001 | 50.5188 | —— | <.0001 |  | -0.00517 | 0.001957 | 6.977103 | —— | 0.0083 |
| hCG_21_^2^ | 9.9800E-09 | 0.0000 | 22.4649 | —— | <.0001 |  | 8.09E-08 | 4.15E-08 | 3.806907 | —— | 0.051 |
| hCG_21_/hCG_14_ | -7.1706 | 2.7906 | 6.6027 | —— | 0.0102 |  | -25.2967 | 11.58429 | 4.768572 | —— | 0.029 |
| (hCG_21_/hCG_14_)^2^ | 1.947011 | 0.6366 | 9.3543 | —— | 0.0022 |  | 5.91132 | 3.610506 | 2.680605 | —— | 0.1016 |
| Cause 2 VS 1 * | 0.2640 | 1.3267 | 0.0396 | 1.30 (0.10-17.54) | 0.8423 |  | 1.857174 | 41.65585 | 0.001988 | 6.41 (0-1.8396E+36) | 0.9644 |
| Cause 3 VS 1 * | 1.9794 | 0.7523 | 6.9222 | 7.24 (1.66-31.63) | 0.0085 |  | -2.44761 | 2.100071 | 1.358365 | 0.087(0.001-5.30) | 0.2438 |
| Cause 4 VS 1 * | 0.6148 | 0.4391 | 1.9605 | 1.85 (0.78-4.37) | 0.1615 |  | -3.09752 | 1.591326 | 3.788874 | 0.05 (0.002-1.02) | 0.0516 |
| Cause 5 VS 1 * | 0.8995 | 0.5173 | 3.0236 | 2.46 (0.89-6.78) | 0.0821 |  | -7.96995 | 7.906744 | 1.016053 | 0 (0-1858.02) | 0.3135 |
| Key: EP, ectopic pregnancy; IUP, intrauterine pregnancy; BCP, biochemical pregnancy; 1, male infertility; 2, endometriosis; 3, annovulatory infertility; 4,tubal factor infertility; 5, unexplained infertility and others；OR，odds ratio. | | | | | | | | | | | |
